# Heterotopia of salivary gland tissue in the pancreas

**DOI:** 10.1186/s13000-023-01385-x

**Published:** 2023-08-30

**Authors:** Sandrina Martens, Katarina Coolens, Catharina Olsen, Pierre Lefesvre, Ilse Rooman

**Affiliations:** 1 Laboratory of Medical and Molecular Oncology, Vrije Universiteit Brussel (VUB), Brussels, Belgium; 2 Present Address: Department of Cardio and Organ Systems, Biomedical Research Institute, Hasselt University, Diepenbeek, Belgium; 3 Brussels Interuniversity Genomics High Throughput Core (BRIGHTcore), VUB-ULB, Brussels, Belgium; 4 Clinical Sciences, Research Group Reproduction and Genetics, Centre for Medical Genetics, Vrije Universiteit Brussel (VUB), Universitair Ziekenhuis Brussel (UZ Brussel), Brussels, Belgium; 5 Interuniversity Institute of Bioinformatics in Brussels (IB)2, VUB-ULB, Brussels, Belgium; 6 Department of Pathology, UZ Brussel, Brussels, Belgium

**Keywords:** Heterotopia, Salivary gland, Submandibular gland, Pancreas, Ectopia

## Abstract

Heterotopia of the salivary gland occurs mainly in the head and neck region of the human body, rarely in regions such as the rectum, but has never been demonstrated in the pancreas. Within a screening effort of pancreatic samples for detecting ΔNp63 expression, we discovered two pancreatic samples from a 35-year-old male showing salivary gland heterotopia. Immunohistochemical stainings were done for markers of healthy and neoplastic salivary glands and showed expression of calponin, CD142 and KRT14 but not of S100p, GFAP or CD117. A PAS-staining and Alcian Blue staining showed the presence of acid mucins. These staining patterns were consistent with non-neoplastic submandibular gland tissue comprised of abundant seromucous glands, basal cells and myoepithelial cells, all features typically absent in the pancreas. Also, no pancreatic islets of Langerhans were detected. We show for the first time that salivary gland heterotopia can occur at the location of the pancreas.

**Supplementary Information:**

The online version contains supplementary material available at 10.1186/s13000-023-01385-x.

## Introduction

Heterotopia, also known as ectopia of healthy tissue, is a congenital aberration where normal tissue is present outside of its normal location, without any physical connection with the organ where this tissue should typically be present. Some heterotopic tissues are more common than others; for example, 2% of the population has a pancreatic heterotopia, i.e. pancreatic tissue being present in another organ [1]. Pancreatic heterotopia is, therefore, one of the most common heterotopias and can be found throughout the gastrointestinal tract [2]. However, heterotopias in the pancreas are very rare, and only one paper reported a heterotopia of gastric mucosa in the pancreas, at the Ampulla of Vater [3].

Rarely, heterotopia of salivary gland tissue can be found, most commonly described in the head and neck region, such as the middle ear, neck, and hypophysis [4]. In the middle ear, this is often referred to as a choristoma, which can lead to hearing loss [5]. However, salivary gland heterotopia can rarely occur in other parts of the body, such as the rectum and colon [6–8]. While the cause is seemingly in embryonic development, it is unclear whether this heterotopia arises from the abnormal development of vestigial structures or an abnormal differentiation of the local tissue [4, 9].

The pancreas and salivary gland are both accessory glands of the digestive system and share many features, containing acinar and ductal structures and producing digestive enzymes in a secretory fluid. However, there are also marked differences, as the salivary gland contains myoepithelial and basal cells, respectively located around the acinar glands and in the basal layer of the ducts. Notably, the pancreas has no myoepithelial cells, and basal cells occur only rarely in larger ducts, as we reported recently [10]. Additionally, some salivary glands, such as the submandibular gland, contain mucous tubules, which are not present in the pancreas. On the other hand, the pancreas is a mixed exocrine and endocrine gland and thus contains endocrine islets of Langerhans, which produce amongst others insulin and glucagon [11].

Here, we describe for the first time heterotopia of salivary gland tissue at the pancreatic location.

## Materials and methods

### Ethical approval

The patient sample was collected by the Department of Anatomopathology in UZ Brussels. The Committee of Medical Ethics - UZ Brussel gave ethical approval for analysis, and samples were obtained through the Central Biobank UZ Brussel (2022-015). For clinical data, ethical approval was provided by Jessa & Wetenschap (f/2022/111).

### Immunohistochemistry

Haematoxylin-eosin staining was done automatically using the Sakura Tissue-Tek Prisma. The Periodic Acid-Schiff (PAS) and Alcian Blue stainings were done manually. Reagents and the associated protocol from Carl Roth were utilised for the PAS staining. For the Alcian Blue stainings, the Alcian Blue solution of pH 2,5 was made by dissolving 1 g of Alcian Blue 8GX (Gurr; Searle Diagnostic) in 97 ml of distilled water and adding 3 ml of glacial acetic acid (UCB). The neutral red stain was made by dissolving 1 g of neutral red (Merck) in 100 ml of distilled water and adding 0,1 ml of glacial acetic acid. In short, slides were deparaffinised and rehydrated, stained for 15’ in the Alcian Blue solution, rinsed for 5’ in tap water, counterstained with the Neutral Red solution for 1’ and then dehydrated, cleared and mounted.

Immunohistochemical stainings were executed on the automated stainer Ventana Benchmark ULTRA. The following antibodies were used: anti-p40, clone BC28 (07394420001, Roche, Switzerland), anti-Calponin-1, clone EP798Y (05435684001), anti-S100P, clone 16/f5 (06523935001), anti-KRT14, clone SP53 (06732429001), anti-KRT7, clone SP52 (05986818001), anti-CD117, clone EP10 (08763909001) and anti-GFAP, clone EP672Y (05269784001).

Immunofluorescent (IF) stainings were done manually. Sections were baked, deparaffinised and rehydrated. Antigen retrieval was performed using citrate buffer (Sigma-Aldrich) in a pressure cooker (Aptum, Antigen-Retriever) for 40 min, and protein block was done using 25% casein block (Thermo Fisher Scientific). Primary antibodies were incubated overnight at 4 °C. Primary antibodies used were directed against ΔNp63 (1/50, ab273135, Abcam), Vimentin (1/100, AB5733, Sigma-Aldrich), E-cadherin (1/50, 610181, BD Transduction), CD142 (1/100, AF2339, RandD), SOX9 (1/1000, ABE2868, Sigma-Aldrich) and KRT5 (1/100, ab52635, Abcam). Next, slides were incubated with a cocktail of secondary antibodies for 1 h. Antibodies used were donkey anti-mouse Alexa Fluor 647 (1/500), anti-rabbit Cy3 (1/500), anti-rabbit Alexa Fluor 488 (1/500), anti-goat Alexa Fluor 647 (1/500) and anti-chicken Alexa Fluor 647 (1/500). After rinsing, slides were mounted with fluorescent mounting medium with Hoechst added (1/500).

All brightfield and fluorescent stainings were scanned using Axioscan Z.1.

### Whole exome sequencing

DNA extraction from FFPE samples (‘pancreas’ and control organ) was performed using Maxwell® RSC 48 instruments and RSC FFPE Plus DNA Kit (Promega). Following quantification on the Qubit 2.0 with the dsDNA BR Assay Kit (Life Technologies), libraries were constructed on 150 ng of input DNA. The KAPA HyperPlus kit (Roche Sequencing) was used according to the manufacturer’s recommendations, with three modifications: (1) enzymatic fragmentation for 20 min at 37 °C, (2) the usage of 15 µM of our in-house designed unique dual indexes (UDI)/ unique molecular identifier (UMI) adapters, (3) a total of 7 PCR cycles were applied. Target enrichment using xGen Exome Hyb Panel v2 probes was performed according to version 5 of the ‘xGen Hybridization Capture of DNA libraries’ instructions (Integrated DNA Technologies). Pre-capture pooling was limited to 2 samples for a total of 2,2 µg pooled library. Final libraries were qualified on the AATI Fragment Analyzer, using the DNF-474 High Sensitivity NGS Fragment Analysis Kit (Agilent Technologies Inc.) and quantified on the Qubit 2.0 with the Qubit dsDNA HS Assay Kit (Life Technologies). Libraries were sequenced 2 × 100 bp on the Illumina NovaSeq 6000 system (Illumina Inc.), with the NovaSeq 6000 S4 Reagent Kit, by denaturing 1 nM libraries. Sufficient reads were generated to accommodate a 1000x coverage.

Reads were aligned to the human reference genome (hg19), and Picard was used to mark the duplicate reads. Post-processing consisted of realignment around insertions/deletions and base quality score recalibration. Variants were called with GATK Mutect2. Variant annotation was conducted with Annovar. The predicted effect was determined with SIFT4G.

## Results

**Fig. 1:**
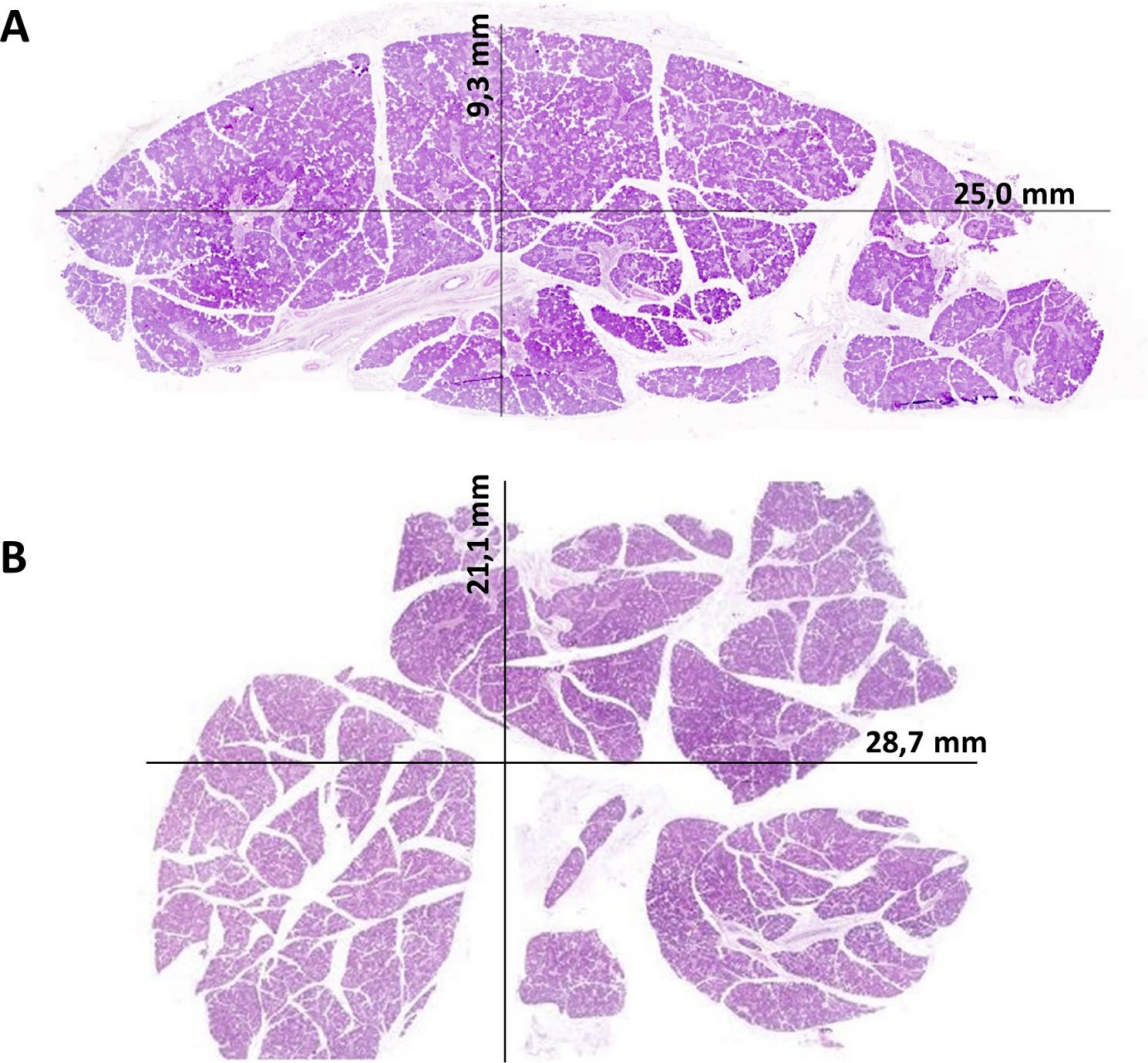
Overview of the two pieces resected from the pancreatic location. A shows a piece of 25 mm x 9,3 mm, while B shows a piece of 28,7 mm x 21,1 mm. Both slides are stained with an HE-staining

Upon our recent discovery of ΔNp63^+^ basal cells in the human pancreas [10], we routinely screened for this marker. We took a particular interest in one patient, a 35-year-old male suffering from vertigo and sciatica, who requested euthanasia. He had previously been treated with a lumbar (L5S1) fusion and lumbar facet joint infiltrations. During the autopsy tissue collection of this patient, two samples of presumed pancreatic tissue were collected, measuring 2,9 cm x 2,1 cm and 2,5 cm x 0,9 cm (Fig. 1A-B). No abnormal macroscopic morphology was noted.

**Fig. 2:**
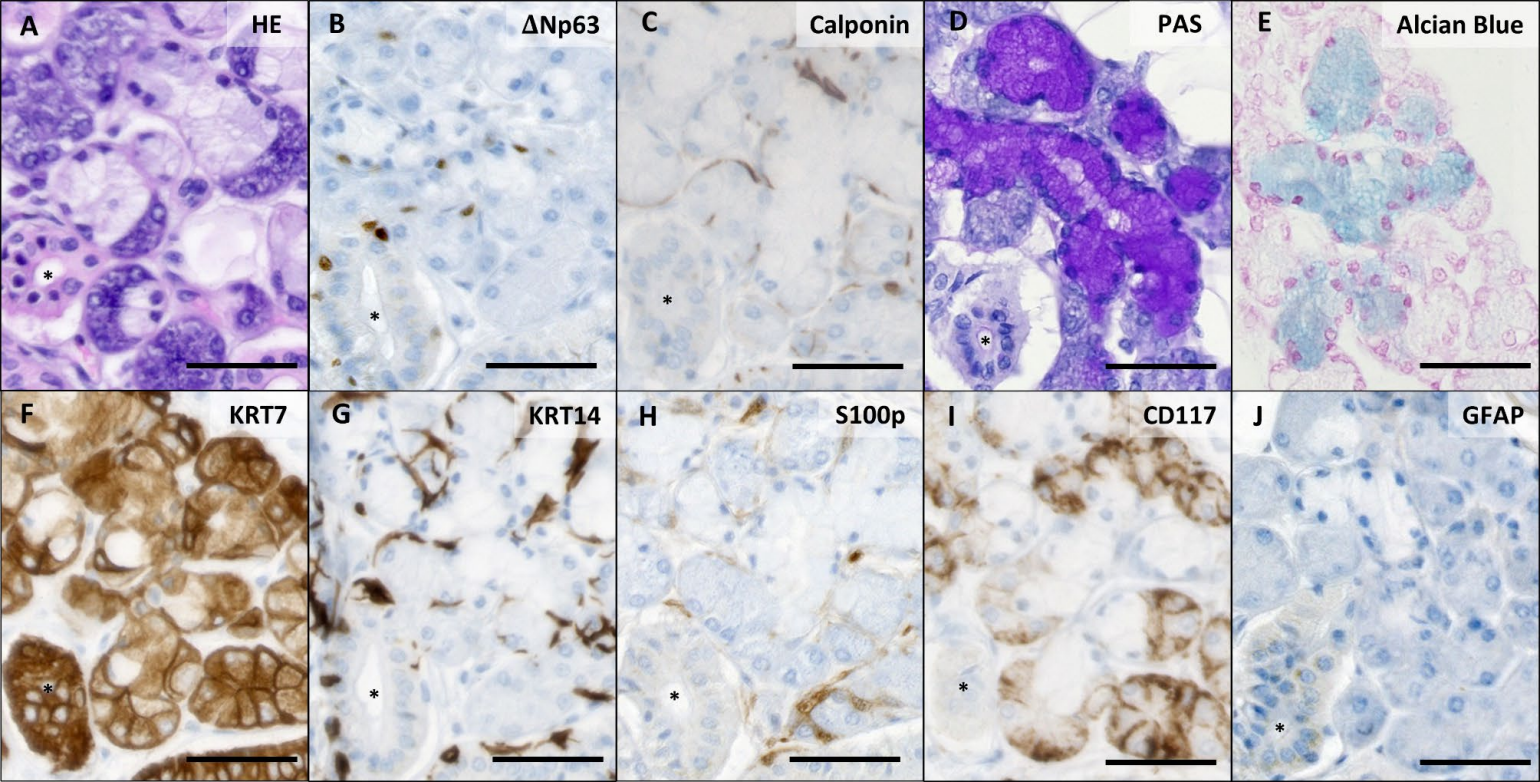
Histology of normal submandibular gland tissue. (A) HE-staining shows ductal tissue (*), serous acinar glands, and seromucous glands. (B) ΔNp63 stains myoepithelial cells in acinar tissue and basal cells in ducts (*). (C) Calponin staining confirms myoepithelial cells in the acinar tissue, while basal cells of the ducts (*) stain negative. (D) PAS staining stains mucus in the seromucous glands. In (E), the presence of acid mucins is confirmed via Alcian Blue staining. (F) KRT7 stains all epithelial cells, while (G) KRT14 stains basal and myoepithelial cells. (H) S100p is generally negative. (I) CD117 expression is low in acinar cells. (J) GFAP is not expressed. Scale bars indicate 50 μm

At the histological level, however, we observed Keratin (KRT)7^+^ serous acinar cells and mucous tubules surrounded by ΔNp63^+^, KRT14^+^, and calponin^+^ myoepithelial cells in which mucous was detected in the PAS and Alcian Blue stainings, which, interestingly, are not typical of pancreatic histology (Fig. 2A-G). Ducts (asterisks in Fig. 2) embedded in connective tissue were present and contained abundant basal cells, characterised by ΔNp63 and KRT14 expression and negative for calponin. The abundance of these cells was remarkable, knowing that basal cells in the pancreas are scarce [10]. S100p was not expressed (except for moderate signal in the connective tissue), CD117 was expressed moderately in the serous acinar cells, and GFAP (Glial Fibrillary Acidic Protein) was completely negative (Fig. 2H-J).

**Fig. 3:**
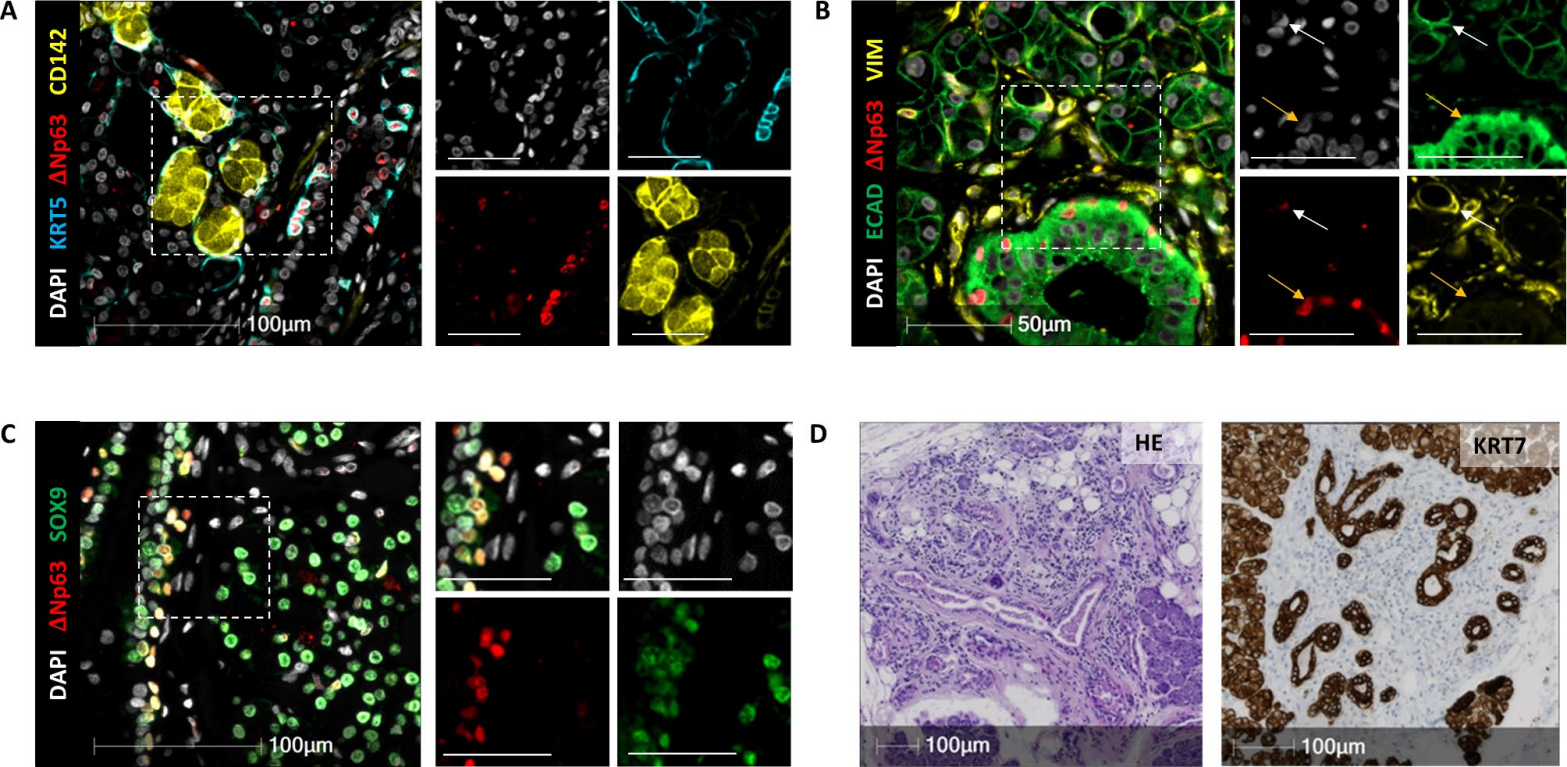
The expression profile of the pancreatic sample is reminiscent of the healthy salivary gland. (A) Staining for KRT5, ΔNp63, and CD142. (B) Staining for ECAD, ΔNp63, and VIM. The full arrow indicates a myoepithelial cell, while the yellow arrow points to a basal cell. (C) Staining for ΔNp63 and SOX9. (D) HE-staining of the atrophic area (left) and KRT7 staining in brown (right) shows mainly duct and stromal tissue

CD142, also known as coagulation factor III, stains the pancreatic basal cells and is present here in the many seromucous glands (Fig. 3A), again atypical of the pancreas. Of note, CD142 is typically present in saliva [12]. The staining pattern could be seen throughout the entire tissue, and no areas with abnormal architecture were detected. Moreover, myoepithelial cells, that are not present in a normal pancreas, were seen surrounding the seromucous glands (Fig. 3B). They expressed vimentin, ΔNp63, and weakly E-Cadherin, while the basal cells did not express vimentin but expressed E-cadherin strongly (Fig. 3B). Notably, the basal cells here express SOX9, just like basal cells in the salivary gland but unlike pancreatic basal cells (Fig. 3C). Some atrophic tissue was apparent in one area, with a loss of KRT7^+^ acinar cells and increased stroma (Fig. 3D).

In summary, the stainings markedly differed from the human pancreas, where ΔNp63 and KRT14-expressing basal cells occur very rarely, and no myoepithelial cells expressing calponin are present [10]. Seromucous glands, which can be seen clearly on the PAS-staining and Alcian Blue staining, also do not exist in the pancreas. On the contrary, these stainings were consistent with the typical salivary gland histology. In agreement, no islets of Langerhans were detected on HE staining, and no expression of pancreatic endocrine islet hormones was detected by immunostaining (not shown).

**Fig. 4:**
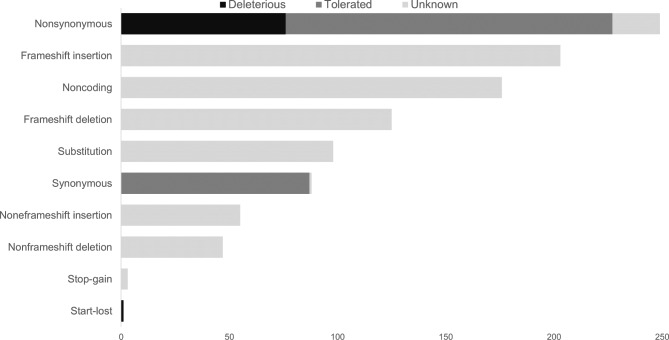
All SNPs detected per category in the heterotopic sample compared to the control tissue. For the mutations of which effect was predicted, deleterious mutations are shown in black and tolerated mutations in dark grey

Whole exome analysis was performed on the patient’s heterotopic tissue and a control organ (Fig. 4). A novel mutation in SHH (Sonic Hedgehog), a gene important in the development of both the pancreas and the salivary gland, was detected. Aside from this, there were also novel mutations in HNF1B and PDX1, both important in the development of the pancreas. Aside from these novel mutations present in the heterotopic sample, we detected previously reported mutations as well. Deleterious mutations were present in ZNF717, a Zinc Finger protein. These mutations have been found in for example oesophageal squamous cell carcinoma (e.g. p.H503P, p.C515S) and colorectal cancer (e.g. R561I). A mutation in the FRG2C gene (R156C) has been found in thyroid neoplasms. However, we did not find any mutations known to be involved in the pancreas or salivary gland pathology.

## Discussion

Until now, heterotopia of salivary gland tissue in the pancreas has not been reported. Since the salivary gland and pancreas share a similar morphology, it may be challenging to discover such heterotopia upon autopsy unless specific stainings are performed.

Since the heterotopia was discovered retrospectively, it is unclear whether it resulted in clinical symptoms in this patient, and it was impossible to investigate the size and extent of this heterotopia. Therefore, we do not know whether the pancreas retained its average size and whether other areas displayed normal pancreatic histology. However, since two pieces resected at random were both salivary gland heterotopia, we can assume that the heterotopic tissue was a large mass. A sampling error can be ruled out, as the size of the pieces combined is larger than a normal submandibular gland, and tissues from the head and neck region are not removed during a regular autopsy. While diseases such as pleiomorphic adenomas can occur in heterotopic tissue of the salivary gland [13], we did not detect any abnormal structures.

In contrast to the more frequently reported salivary gland heterotopia in the head and neck region [4, 5], this is the first incidental report in the pancreatic location. In the gastrointestinal tract, rare heterotopia of the salivary gland has been described mainly in the rectum and very rarely in the jejunum, colon, oesophagus, and gastro-oesophageal junction [7]. However, no heterotopias have been described in the literature for accessory glands such as the pancreas. This may be because, with global colorectal screening efforts, masses are more easily detected in the GI tract, while pancreas imaging does not frequently take place.

It is difficult to deduce how this heterotopic tissue may have occurred, as still debated in the literature [4, 9]. It is even unclear whether the submandibular gland has an ectodermal or endodermal origin, while the pancreas is definitely of endodermal origin [14, 15]. Interestingly, it has been shown that salivary gland progenitor cells can differentiate to (endocrine) pancreatic cells in vitro [16], suggesting lineage relationships that likely can extend to the exocrine pancreas lineage. The exocrine pancreas notably has many features in common with the salivary gland regarding gene expression, anatomy, and function, with both glands being organised into acini and ducts and producing digestive enzymes [11, 17]. If both tissues are of endodermal origin, abnormal development of the endodermal lineage may have occurred on the pancreatic site, resulting in heterotopic salivary tissue.

The SNPs detected in the heterotopic tissue were not yet reported in the salivary gland or pancreatic diseases. However, some SNPs were already reported in neoplasms of other organs. Therefore, the reported list of SNPs warrants more future research, specifically in the context of salivary and pancreatic cell differentiation and that of their progenitors in the endodermal lineage, as speculated above. While initial data can be collected on the impact of the SNP on the protein function, promising leads can be investigated in genetically engineered mice for assessing the resulting phenotype. Unravelling their function can reveal their potential role in the development of diseases and may shed light on clinical consequences.

In conclusion, upon autopsy, it is warranted that the pancreas and its surrounding are vigorously investigated for a similar heterotopia that could easily be missed. More research should investigate the causes of salivary gland heterotopia, including now that at the pancreatic location.

### Electronic supplementary material

Below is the link to the electronic supplementary material.

Supplementary Material 1
